# A nationwide postal questionnaire survey: the presence of airway guidelines in anaesthesia department in Sweden

**DOI:** 10.1186/1471-2253-14-25

**Published:** 2014-04-07

**Authors:** Kati Knudsen, Ulrika Pöder, Marieann Högman, Anders Larsson, Ulrica Nilsson

**Affiliations:** 1 Department of Public Health and Caring Sciences, Caring Sciences, Uppsala University, Box 564, Uppsala, SE 751 22, Sweden; 2 Department of Health and Caring Sciences, University of Gävle, Gavle, SE 801 76, Sweden; 3 Department of Medical Sciences, Respiratory Medicine and Allergology, Uppsala University, Uppsala, SE 751 85, Sweden; 4 Centre for Research & Development, Uppsala University/ County Council of Gävleborg, Gavle, SE 801 88, Sweden; 5 Department of surgical sciences, Uppsala University, Anaesthesiology & ICM, Uppsala SE 751 85, Sweden; 6 School of Health and Medical Sciences, Örebro University, Örebro SE 701 82, Sweden

**Keywords:** Airway guidelines, Airway management, Patient safety

## Abstract

**Background:**

In Sweden, airway guidelines aimed toward improving patient safety have been recommended by the Swedish Society of Anaesthesia and Intensive Care Medicine. Adherence to evidence-based airway guidelines is known to be generally poor in Sweden. The aim of this study was to determine whether airway guidelines are present in Swedish anaesthesia departments.

**Methods:**

A nationwide postal questionnaire inquiring about the presence of airway guidelines was sent out to directors of Swedish anaesthesia departments (n = 74). The structured questionnaire was based on a review of the Swedish Society of Anaesthesia and Intensive Care voluntary recommendations of guidelines for airway management. Mean, standard deviation, minimum/maximum, percentage (%) and number of general anaesthesia performed per year as frequency (n), were used to describe, each hospital type (university, county, private). For comparison between hospitals type and available written airway guidelines were cross tabulation used and analysed using Pearson’s Chi-Square tests. A p- value of less than 0 .05 was judged significant.

**Results:**

In total 68 directors who were responsible for the anaesthesia departments returned the questionnaire, which give a response rate of 92% (n 68 of 74). The presence of guidelines showing an airway algorithm was reported by 68% of the departments; 52% reported having a written patient information card in case of a difficult airway and guidelines for difficult airways, respectively; 43% reported the presence of guidelines for preoperative assessment; 31% had guidelines for Rapid Sequence Intubation; 26% reported criteria for performing an awake intubation; and 21% reported guidelines for awake fibre-optic intubation. A prescription for the registered nurse anaesthetist for performing tracheal intubation was reported by 24%. The most frequently pre-printed preoperative elements in the anaesthesia record form were dental status and head and neck mobility.

**Conclusions:**

Despite recommendations from the national anaesthesia society, the presence of airway guidelines in Swedish anaesthesia departments is low. From the perspective of safety for both patients and the anaesthesia staff, airway management guidelines should be considered a higher priority.

## Background

Recent studies have shown that complications due to airway manipulations are uncommon in anaesthesia practice, but when they occur they may be deleterious [1,2]. In order to promote safe, evidence-based practice, standards or clinical guidelines are developed. Guidelines allow greater user flexibility compared with standards [3] and are defined as “systematically-developed evidence-based statements to assist providers, recipients, and other stakeholders in making informed decisions about appropriate health interventions” (p 2,WHO 2003) [4]. International and national guidelines can be locally modified, and local guidelines can be developed. Guidelines cannot guarantee any specific outcomes and are therefore not absolutely required steps. However, soundly developed guidelines should be seen as a summary of good practice, and side steps from such protocols for the benefit of the individual patient should not be discouraged without motivations [3]. Non-adherence to guidelines has been explained by the fact that guidelines are designed for the average patient. Clinicians might be less enthusiastic about standard regimens, as no two patients are exactly alike. Among the most prominent reasons for not following guidelines is the lack of a peer-reviewed evidence-base [5]. However, adherence to evidence-based airway guidelines is generally described as poor as well [6].

Worldwide, anaesthesia societies have designed their own airway guidelines to achieve safe airway procedures, e.g. for endotracheal intubation and extubation [7-9]. Such guidelines consist of an assessment of the airway, adequate airway equipment, and a detailed plan of how to handle a failed airway [10]. Also, it is stated in many of these guidelines that a systematically performed airway evaluation should include written documentation of individual details of the patients’ airway [11,12]. We strongly believe that this is a key point in the practice of anaesthesia to further improve patient safety. In Sweden, airway management guidelines have been recommended by the Swedish Society of Anaesthesia and Intensive Care Medicine (SFAI) [13]. Furthermore, in Swedish health care policies, health care staff are to provide conditions that promote patients to participate in everyday health care interactions to ‘provide individually adjusted information’, ‘the possibility to choose between different treatments alternatives’, and ‘the possibility for a second opinion’ [14]. Using guidelines for clinical practice can help the anaesthesia staff with their decision making and promote patient participation. The aim of this study was to explore the presence of airway guidelines in Swedish anaesthesia departments.

## Methods

Data for this study was obtained from anaesthesia departments in Sweden (N = 74), which were identified by the Swedish Association of Local Authorities and Regions [15]. In March 2011, a questionnaire was sent out to all directors of Swedish anaesthesia departments. The director or assistant director was asked to complete and return the questionnaire in a prepaid envelope. Postal reminders were sent out twice. Those who did not respond within three months of the second reminder (n = 14) were reminded a third time by telephone. Information about the study and an informed consent form were sending with the questionnaire and the informed consent was returned with the questionnaire. According to the Swedish Act on the Ethical Review of Research, formal ethical approval was not required for this study since the response to this survey does not include any sensitive information about patients. However, written informed consent was received from each responding director by ensuring confidentiality and voluntary participation in the study. All data were collected and treated confidentially to avoid identifying a specific anaesthesia department.

The structured questionnaire was constructed for the purpose of the study, and was based on a review of the SFAI recommendations and guidelines for airway management [13]. The questions were drafted by the first author then discussed in detail within the research group. The questionnaire’s appropriateness was thereafter evaluated by an expert in clinical guideline development in order to improve the clarity of the questions before the questionnaire was sent out. The questionnaire included two questions about hospital category and the number of general anaesthesia procedures per year, along with eight other questions about whether the department had 1) an airway algorithm, 2) written patient information in case of difficult airway, 3) guidelines for difficult airway, 4) guidelines for preoperative airway assessment, 5) guidelines for Rapid Sequence Intubation (RSI), 6) guidelines for criteria to perform awake intubation, 7) instructions for an awake fibre-optic intubation, and 8) a prescription for when a registered nurse anaesthetist (RNA) or anaesthesiologists should perform endotracheal intubation. The questions were answered in a tick-box format as “Yes” or “No”. If the answer was “Yes”, the respondent was asked to send back copies of the guidelines that they used. In addition, the respondent was asked to include the department’s anaesthetic record form in order to review whether a fill-out “box” of airway evaluation and planning was pre-printed.

### Statistical analysis

Data were entered and analysed using descriptive statistics computed in PASW Statistics 20.0 for Windows (SPSS Inc. an IBM Company, Chicago, IL, USA). Descriptive statistics were computed for all variables and were described using means and standard deviations (SD) for continuous variables and frequencies and percentages for categorical variables. The presence of written guidelines for airway management (Yes or No) between the anaesthesia department categories (university, county, private) were analysed using Pearson Chi-Square independent 2-tailed test. Fishers’ exact test was used in case an expected count was less than five in one or more cells. A p < 0.05 was considered statistically significant.

## Results

Out of the 74 questionnaires sent out, 68 replies were received (92% response rate). Of the anaesthesia departments that responded, 53 were from county hospitals, nine from university hospitals, and six from private hospitals. The number of general anaesthesia procedures performed per year range from minimum 500 to maximum 28,000. Most number of general anaesthesia procedures/year was performed by university hospitals (14,389 ± 7,674), followed by county hospitals (5,646 ± 4,143), while private hospitals stand for minority procedures per year (4,063 ± 1,837). Of the departments, 68% reported the presence of guidelines for an airway algorithm, 52% reported difficult airway management guidelines, and 52% reported written information for patients in case they have a difficult airway. Guidelines for awake fibre-optic intubation were reported by only 14 (21%) departments. There was no statistically significant difference in the proportion of reported written guidelines for airway management between type of hospitals, except for guidelines for difficult airway (p = 0.049). Eight of the nine university hospitals reported guidelines for difficult airway compared with only 23 of 53 county and 2 of 6 private hospitals. The percentage of available written guidelines for airway management within anaesthesia departments is summarised in Table 1.

**Table 1:** Type of hospital and presence of airway management guidelines

**Type of guidelines**	**Total (n =68)**		**County hospital (n = 53)**		**University hospital (n = 9)**		**Private hospital (n = 6)**	
	**Yes n (%)**	**No n (%)**	**Yes n (%)**	**No n (%)**	**Yes n (%)**	**No n (%)**	**Yes n (%)**	**No n (%)**
Guidelines for an airway algorithm	46 (68)	22 (32)	35 (66)	18 (34)	8 (88)	1 (11)	3 (50)	3 (50)
Guidelines for difficult airway*	35 (52)	33 (48)	25 (47)	28 (53)	8 (88)	1 (12)	2 (33)	4 (67)
Written patient information (anaesthesia problem card) in case of difficult airway	35 (52)	33 (48)	30 (56)	23 (44)	3 (33)	6 (67)	2 (33)	4 (67)
Guidelines for preoperative assessment	29 (43)	39 (57)	22 (42)	31 (58)	3 (33)	6 (67)	4 (67)	2 (33)
Guidelines for Rapid Sequence Intubation	21 (31)	47 (69)	16 (30)	37 (70)	3 (33)	6 (67)	2 (33)	4 (67)
Guidelines for criteria to perform awake intubation	18 (26)	50 (74)	15 (28)	38 (72)	3 (33)	6 (67)	0 (0)	6 (100)
Prescription to RNA to perform the tracheal intubation	16 (24)	52 (76)	14 (26)	39 (74)	2 (22)	7 (78)	0 (0)	6 (100)
Guidelines for awake fibre-optic intubation	14 (21)	54 (79)	10 (19)	43 (81)	4 (45)	5 (55)	0 (0)	6 (100)

*Fishers’ exact test, p = 0.049 between type of hospitals.

RNA, registered nurse anaesthetist.

In total, 214 guidelines were reported, of which 132 (67%) were not verified by an attachment. Forty six (21%) of the attached guidelines were those recommended by the SFAI, and 36 (17%) of the attached guidelines were developed by the local departments. Of the 46 departments that reported having a guideline for an airway algorithm, 11 attached locally developed guidelines and nine the SFAI airway guidelines, whereas the remaining 26 did not attach any guidelines. Among the 35 departments that reported giving written postoperative information to the patient in case of a difficult airway, 12 attached a guideline. In all cases, an anaesthesia problem card was attached, as recommended by the SFAI. Fourteen departments reported having a guideline for awake fibre-optic intubation and one sent their locally developed guidelines (Table 2).

**Table 2:** Presence of guidelines for airway management within anaesthesia departments (n = 68)

	**n (%)**	**Developed by department**	**Recommendations according to SFAI**	**No guidelines sent**
		**n (%)**	**n (%)**	**n (%)**
Guidelines for an airway algorithm	46 (68)	11 (24)	9 (20)	26 (56)
Written patient information (anaesthesia problem card) in case of difficult airway	35 (52)	0 (0)	12 (34)	23 (66)
Guidelines for difficult airway	35 (52)	0 (0)	17 (49)	18 (51)
Guidelines for preoperative assessment	29 (43)	9 (31)	0 (0)	20 (69)
Guidelines for Rapid Sequence Intubation	21 (31)	9 (43)	0 (0)	12 (57)
Guidelines for criteria to perform awake intubation	18 (26)	3 (17)	8 (44)	7 (39)
Prescription for RNA to perform the tracheal intubation	16 (24)	3 (19)	0 (0)	13 (81)
Guidelines for an awake fibre-optic intubation	14 (21)	1 (7)	0 (0)	13 (93)

SFAI, Swedish Society of Anaesthesiology and Intensive Care Medicine; RNA, registered nurse anaesthetist.

Documents sent that did not match the guideline criteria for this study are presented as follows: three directors sent user manuals with photographic images depicting how to perform fibre-optic bronchoscopy and a list of the equipment on their airway trolley; one respondent attached an abstract and photocopies from an article in the Swedish medical journal for physicians that was used as a guideline [16]. Twenty departments did not send any guidelines for preoperative assessment, but two of these departments stated that they used the SFAIs guidelines and two reported using Mallampati’s test, Thyromental distance, and an examination of the mouth and dental status.

Fifty one departments sent an anaesthesia record form (Table 3). In the airway “box” in the anaesthesia record, dental status and head and neck mobility were the most frequently pre-printed elements followed by the Cormack & Lehanes grade. The least commonly reported box was sternomental distance and the number of intubations attempted.

**Table 3:** List of the most commonly pre-printed airway elements in the anaesthesia record collected (n = 51)

	**Yes (%)**	**No (%)**
Tooth status	35 (69)	16 (31)
Head and neck mobility	19 (37)	32 (63)
Mallampati test	17 (33)	34 (67)
Mouth opening	17 (33)	34 (67)
Thyromental distance	13 (25)	38 (75)
Sternomental distance	4 (8)	47 (92)
Cormack & Lehanes grade	23 (45)	28 (55)
Mask ventilation	13 (25)	38 (75)
Number of intubations attempt	6 (12)	45 (88)
Evaluation of extubation	26 (51)	25 (49)

## Discussion

We surveyed the presence of airway management guidelines in Swedish anaesthesia departments and found an important lack of guidelines. In those cases when the departments sent their guidelines, many were developed nationally by SFAI and few local guidelines were present. Our findings shows that about half of the departments reported the presence of guidelines for an airway algorithm, a written patient information card in case of difficult airway, and guidelines for difficult airway, but less than half reported the presence of guidelines for preoperative assessment. A third or less reported a guideline for Rapid Sequence Intubation, criteria for performing an awake intubation, or a prescription for the RNA to perform a tracheal intubation. The most frequently pre-printed preoperative elements in the anaesthesia record form were dental status and head and neck mobility.

A lack of airway guidelines was not unexpected; similar findings have been described in other countries [11,17]. Even if guidelines aim to improve safe practices and to promote patient participation [14,18], existing guidelines for airway management are not always used [1]. An airway algorithm was reported as being present in 68% of the departments. This high number of reported guidelines for an airway algorithm could perhaps be explains by the fact that the SFAI has recently developed a new airway algorithm [13]. On the other hand, only one third of the departments had written guidelines for Rapid Sequence Intubation. This is interesting since this method is one of the most commonly discussed and questioned induction techniques when unanticipated difficult airway occurs, and requires training and experience [19]. Furthermore, only about half of the departments had a defined algorithm for the management of a difficult airway. Although the purpose of many of these airway guidelines is safer airway management, relatively little consideration has been given to their usefulness. More could be done to implement such guidelines locally [20]. In Europe; many anaesthesia societies collaborate and share guidelines for clinical procedures. Such collaborations support the work of anaesthesia professionals as they contribute to high-quality care, and also reduce practice variation from one country to another, as well as reduce the variety of practices among local hospitals [21,22].

In accordance with recommendations from the SFAI, written information (anaesthesia problem card) was the most adopted guideline by most of the departments. In case of difficult airway, SFAI recommends that the anaesthesia staff inform the patient orally, but also by use of a written card, with the recommendation that this card be shown in case of a new anaesthesia. However, only half of the departments gave written information in the event of a difficult airway. This contradicts the regulations of the Swedish Patient Safety Act (2010:659), which states that patients are entitled to be involved in decisions that affect them [14]. This regulation is in line with the World Health Organization’s definition of guidelines; i.e. that they should promote patients’ ability to participate in decision-making regarding their own care [4]. In order to facilitate a shared decision-making preoperative airway assessment, preparation and planning should always be performed in consultation and dialogue with the patient. The patient should also be informed about the planned approach for anaesthesia and the risk factors. In our study, less than half of the hospitals reported the presence of guidelines for preoperative airway assessment. Additionally, one half of the anaesthesiologists in a European survey did not perform preoperative airway tests, despite existing guidelines [23]. Also, McPherson et al. [24] described that anaesthesiologists did not always perform a preoperative airway assessment.

Even if airway guidelines are available that describe how to manage a difficult airway, it seems that not all professionals follow these directions. We speculate that our finding of the lack of guidelines could depend on an unwillingness to develop standardised guidelines for otherwise individually planned patient care [5]. Lack of time and perhaps also a lack of knowledge about how to create evidence-based guidelines could be other possible explanations. Since guidelines are not absolute requirements in the health care organisation, the development of guidelines might not be a prioritised task. However, the absence of guidelines may jeopardise safe practice and thereby a favourable patient outcome [3]. Preoperative airway assessment should not depend on the individual anaesthetists’ skill and knowledge [25].

In the present study, deficiencies in pre-printed documentation of airway elements in the anaesthesia records were observed; for example, for mask ventilation and the number of intubation attempts. These clinically important variables have previously been associated with difficult airway; [26] therefore, routinely documenting this is suggested. Furthermore, inadequate preoperative airway documentation has been shown to increase adverse airway events during anaesthesia [27] and standardised documentation of airway variables in anaesthesia records have been identified as important for patient safety [28,29]. From our point of view, pre-printed “box-plots” for airway management in the anaesthesia record are useful, and could lead to less variability of how to evaluate the patients’ airway. However, this survey did not explore this issue.

The anaesthesia professionals’ competence may differ between European countries [30]. In Sweden, anaesthesiologists usually perform the preoperative airway assessment, whereas RNAs perform and maintain the anaesthesia according to specified protocols and agreements [31]. Only a third of departments reported the presence of guidelines for when anaesthesiologists were allowed to delegate the tracheal intubation to a RNA. One explanation could be that RNAs in Sweden are educated in and have a long tradition of providing anaesthesia to patients and therefore no guidelines are requested. Thus, RNAs in Sweden are qualified and well trained to perform endotracheal intubations independently on patients without the direct supervision of anaesthesiologists [31]. However, from RNAs’ point of view, using airway scores that can predict easy tracheal intubation are warranted in order to better decide the right competence level and profession for intubating the individual patient [32].

An important strength of our study is the high response rate. A limitation of the study is the possibility of a false negative or positive picture because an electronic copy of the guidelines was not always returned with the questionnaire. In those cases where a guideline was reported as being present, we do not know how well it was adhered to. However, similar findings are described from other countries [11,17]. Moreover, the results of the present study also reflect our clinical experience indicating that the presence of airway guidelines can be improved. Our purpose was to explore and describe the presence of airway guidelines in anaesthesia departments in Sweden. The availability of such guidelines was not considered a high priority in our departments. We did not attempt to assess the quality of the guidelines returned to us, i.e. if the guidelines were systematically developed and evidence-based. This topic needs further investigation because updated evidence-based guidelines describing current best practices could contribute to adherence to guidelines [5].

## Conclusions

Despite recommendations from the national anaesthesia society, the presence of airway guidelines in Swedish anaesthesia departments is low. From the perspective of safety for both patients and anaesthesia staff, development and use of updated evidence-based guidelines for airway management should be considered a higher priority.

## Abbreviations

SFAI: Swedish Society of Anaesthesia and Intensive Care Medicine; RSI: Rapid sequence intubation; RNA: Registered nurse anaesthetist; SD: Standard deviations.

## Competing interests

None of the authors have any competing interest associated with this study.

This study was financial supported in part by the Department of Public Health and Caring Sciences, Caring Sciences, Uppsala University and University of Gävle, Faculty of Health and Occupational Studies, Department of Health and Caring Sciences, Gävle and Centre for Research & Development, Uppsala University/County Council of Gävleborg, Gävle, Sweden.

The Editorial services were provided by San Francisco Edit (http://www.sfedit.net), assists with the language edition of the manuscript.

Preliminary data for this study were presented as a poster presentation at the Nordic Congress for Anaesthesia and Intensive Care Nurses (NOKIAS, 19–21 September 2013, Copenhagen).

## Authors’ contributions

KK and UN design of the study and drafted the manuscript. KK sent the questionnaires to participated directors, performed the statistical analysis and wrote the manuscript. MH, AL, UP, and UN analysed the statistics and edited the manuscript critically. All authors read and approved the final manuscript.

## Authors’ information

Marieann Högman, Anders Larsson, Ulrika Pöder and Ulrica Nilsson are co-authors.

## Pre-publication history

The pre-publication history for this paper can be accessed here:

http://www.biomedcentral.com/1471-2253/14/25/prepub
